# Reliable RANSAC Using a Novel Preprocessing Model

**DOI:** 10.1155/2013/672509

**Published:** 2013-02-20

**Authors:** Xiaoyan Wang, Hui Zhang, Sheng Liu

**Affiliations:** ^1^School of Computer Science and Technology, Zhejiang University of Technology, Hangzhou 310023, China; ^2^College of Information Engineering, Zhejiang University of Technology, Hangzhou 310023, China

## Abstract

Geometric assumption and verification with RANSAC has become a crucial step for corresponding to local features due to its wide applications in biomedical feature analysis and vision computing. However, conventional RANSAC is very time-consuming due to redundant sampling times, especially dealing with the case of numerous matching pairs. This paper presents a novel preprocessing model to explore a reduced set with reliable correspondences from initial matching dataset. Both geometric model generation and verification are carried out on this reduced set, which leads to considerable speedups. Afterwards, this paper proposes a reliable RANSAC framework using preprocessing model, which was implemented and verified using Harris and SIFT features, respectively. Compared with traditional RANSAC, experimental results show that our method is more efficient.

## 1. Introduction 

Feature matching is a basic problem in computer vision. Corresponding to local features has become the dominant paradigm for structure from motion [1, 2], image retrieval [3], and medical image processing [4]. It is a crucial issue to correspond to the features accurately and efficiently [5, 6]. Most applications are built upon a general pipeline consisting of steps for extracting features from images, matching them to obtain correspondences, and applying some forms of geometric verification to reject the outliers. The geometric verification is extremely critical for the pipeline's success. It has been proven that RANSAC [7] is the best method of choice for this pipeline [8]. However, there are two obvious shortcomings in RANSAC processing. On one hand, it is time-consuming. On the other hand, when the sampling time is restricted artificially, the selected matching pairs may not be correct. 

Consequently, numerous extensions for RANSAC have been proposed to speed up different RANSAC stages, such as SCRANSAC [8], optimal randomized RANSAC [9], and other improved methods [10–12]. However, even with these extensions, the geometric verification is still a major bottleneck in applications. In addition, most of the improved methods cost considerable implementation runtime and are difficult to tune for optimal performance.

This paper proposes a fast and simple RANSAC framework based on a preprocessing model. It can result in a reduced correspondence set with a higher inlier percentage, on which RANSAC will converge faster to a correct solution. This model can successfully acquire a subset *E* with higher probability being inliers from the initial corresponding set *P*. Then, a reliable fundamental matrix **F** or a homography matrix **H** can be estimated from subset *E*. Owing to *E* with higher inliers ratio, the estimated **H** or **F** is more reliable. Finally, the outliers in set *P* can be rejected according to the estimated **H** or **F**. Comparing with other improved methods, the proposed approach in this paper can achieve similar speedup while being considerably simpler to implement. 

The rest of this paper is organized as follows. In Section 2, this paper discusses RANSAC for outlier rejection and introduces preprocessing model, including its motivation and algorithm flowchart. In Section 3, a novel RANSAC framework based on Preprocessing Model is proposed.  Section 4 presents the experimental results and data analysis. The last part is a summarization of this paper.

## 2. Outlier Rejection

RANSAC has become the most popular tool to solve the geometric estimation problems in datasets containing outliers, which was first proposed by Fischler and Bolles in 1981 [7]. It is a nondeterministic algorithm with a purpose that it can produce a reasonable result only with a certain probability. 

### 2.1. RANSAC

RANSAC operates in a hypothesized-and-verified framework. The basic assumption of RANSAC algorithm is that the data consists of “inliers”, that is, the data whose distribution can be explained by some set of model parameters. And “outliers” are the data which do not fit the model. The outliers probably result from errors of measurement, unreasonable assumptions, or incorrect calculations. RANSAC randomly samples a minimal subset *s* of size from the initial set in order to hypothesize a geometric model. This model is then verified against the remaining correspondences, and the number of inliers, that is, of correspondences consistent with the model, is determined as its score. RANSAC achieves its goal by iteratively selecting a random subset of the original data, which are hypothetical inliers. This procedure is iterated until a certain termination criterion is met. In confidence *p*, ensure that at least one sampling within *N* times sampling, the elements are all inliers. The equation is

(1) $$\begin{array}{c} N=\frac{\log\left(1-p\right)}{\log\left(1-\varphi^{s}\right)}, \end{array}$$

where *s* is the mean of the minimal size of sampling subset to hypothesize the geometric model, and *φ* represents the probability of a point being an inlier.

The iteration ensures a bounded runtime as well as a guarantee on the quality of the estimated result. As mentioned above, there are some limits in RANSAC processing. Time-consuming is the most urgent problem, especially when the initial inliers rate is low. Hence, this paper proposes a novel RANSAC framework with a preprocessing model to improve it.

### 2.2. Preprocessing Model

The main effort of this preprocessing model is to explore a reduced set with reliable correspondences from initial matching dataset and estimate the geometric model. This model can be divided into the following two steps.

#### 2.2.1. Selecting Reliable Corresponding Pairs

When verifying hypotheses in RANSAC, the corresponding pairs are categorized into inliers and outliers. Since the number of samples taken by RANSAC depends on the inlier ratio, it is desirable to reduce the fraction of outliers in the matching set. Selecting a reduced set with higher inlier ratio is the first step of this preprocessing model. Our approach is motivated by the observation that extracting and exploring a subset *E* with higher probability being inliers is an efficacious idea to improve the runtime of RANSAC. The idea underlying the preprocessing model is to use relaxation technique [13] to acquire a reduced set of more confident correspondences. It leads to a significant speedup of the RANSAC procedure for two reasons. First, RANSAC only needs to operate on a substantially smaller set *E* for verifying model hypotheses. Second, the additional constraints enforced in relaxation method lead to an increased inlier ratio in reduced set *E*. This directly affects the number *N* of iterations. Hence, the preprocessing model converges faster to a correct solution. 

#### 2.2.2. Fundamental Matrix *F* Estimation

Zhang et al. [13] used LMedS technique to discard false matches and estimate fundamental matrix. However, when the inlier ratio is less than 50%, the result estimated by LMedS method may be unreliable. RANSAC is one of the robust methods for fundamental matrix estimation, which can obtain robust result even when the outlier ratio is more than 50%.

**Figure 1:**
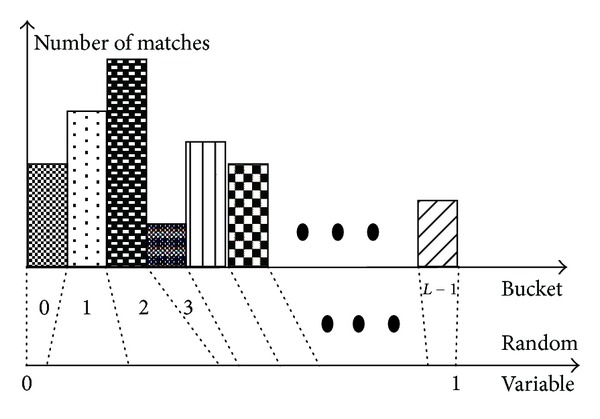
Monte Carlo sampling method.

**Figure 2:**
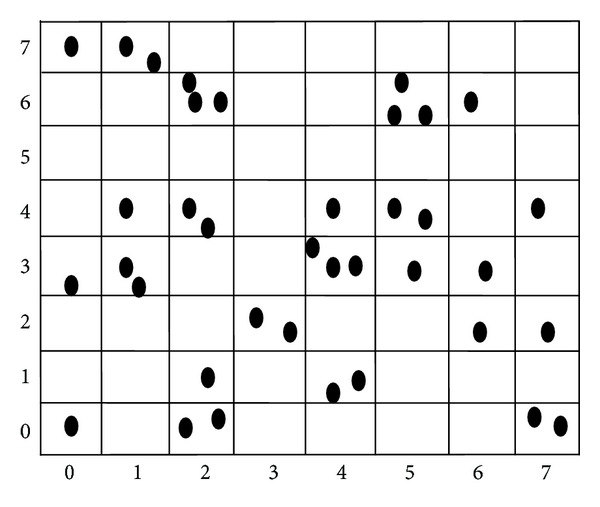
Bucketing technique.

RANSAC is a stochastic optimization method, whose efficiency can be improved by Monte Carlo sampling method [14]. This method is shown in Figure 1. However, the sampling results may be very close to each other. Such a situation should be avoided because the estimation result may be instable and useless. The bucketing technique [14] is used to achieve higher stability and efficiency, which is shown in Figure 2. It works as follows. The min and max of the coordinates of the points are calculated in the first image. The region of the image is then divided into *b* × *b* buckets (shown in Figure 2). To each bucket is attached a set of feature points, and indirectly a set of correspondences, which fall into it. Those buckets which have no matches attached are excluded. In order to estimate fundamental matrix *F*, a subsample of 8 points should be generated. It is selected in 8 mutually different buckets, and then one match in each selected bucket is randomly selected.

Therefore, the fundamental matrix *F* can be estimated accurately and efficiently. This is the second step of the preprocessing model.

## 3. RANSAC Framework with Preprocessing Model

An improved RANSAC algorithm with preprocessing model is proposed in this section. This model can be easily integrated into the RANSAC procedure. The main idea is to suppose knowing some matching pairs being inliers with high probability, which are put into subset *E*  (*E* ⊂ *P*). Therefore, if RANSAC operates in subset *E* with the same confidence, it can calculate closer to the correct fundamental matrix **F** (or homography matrix **H**) with much less time of iteration. Thus, the preprocessing model can achieve the speedups in the whole RANSAC procedure. The steps of our framework are described as in Algorihm 1.

In Algorithm 1,  q* is the threshold of relaxation iteration. In this paper, *q* is set to 60. *p*_0_ is the RANSAC threshold parameter, which is usually set to 95%. Let *φ*_red_ denote the ratio of inliers to all correspondences in set *E*. Then, the probability *p* that in *N* steps RANSAC ensures that at least one sampling within times *N* sampling, the elements are all inliers follow as *p* = 1 − (1 − *φ*_red_^*s*^)^*N*^. Once matrix **F** is obtained in set *E*, we can additionally compute the hypothesis's support on the whole set *P*. In our experiments, we however only perform this last step to report the inlier numbers.

## 4. Experiment and Analysis

In the following, this paper experimentally evaluates the improved RANSAC and compares it with a classical approach. As we know, Harris and SIFT features are most commonly used in correspondence [15, 16]. In order to evaluate an approach comprehensively, choose both Harris and SIFT feature in initial corresponding. The environment of the experiments is Matlab R2010. Computer configuration is 2.10 G (CPU) and 4.00 G (RAM). The experimental images in this paper are from open databases: Visual Geometry Group, Peter Kovesi's home page, and the internet.

### 4.1. Experiment Based on Harris Feature

In the experiments based on Harris feature, this paper chooses match-by-correlation algorithm to obtain the initial matching set *P*. Then, the proposed RANSAC framework is operated on set *P*. The consequent of the Preprocessing Model directly determines the effect of the whole procedure. The reliable set *E* can be acquired by adjusting the model parameters. 

**Figure 3:**
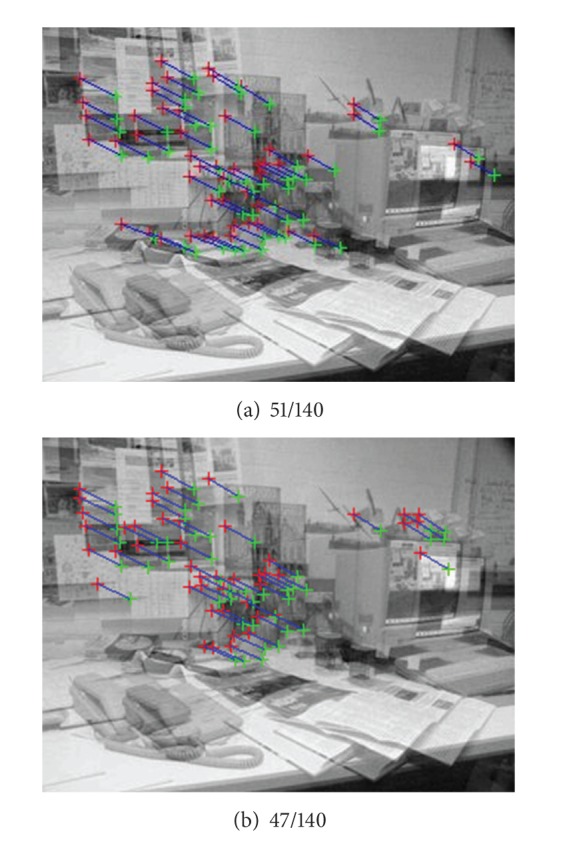
Comparison between our proposed RANSAC and traditional RANSAC.

Figure 3 is the comparison between our approach and the traditional RANSAC.  Figure 3(a) shows the matching result calculated by our improved RANSAC. The result of traditional RANSAC method in the same experimental condition is shown in Figure 3(b). The numbers of iterations in Figures 3(a) and 3(b) are 260 and 361, respectively. 51/140 means extracting 51 inliers from 140 initial putative matching set. From the comparison, it is obvious that the result of our approach is better. The most important is that the iteration times are reduced. Thus, it can improve the runtime of RANSAC successfully. Compared with other improved RANSAC algorithms, our RANSAC framework can achieve the same result, while it is simpler to implement and the sampling times are reduced.

### 4.2. Experiment Based on SIFT Feature

**Figure 4:**
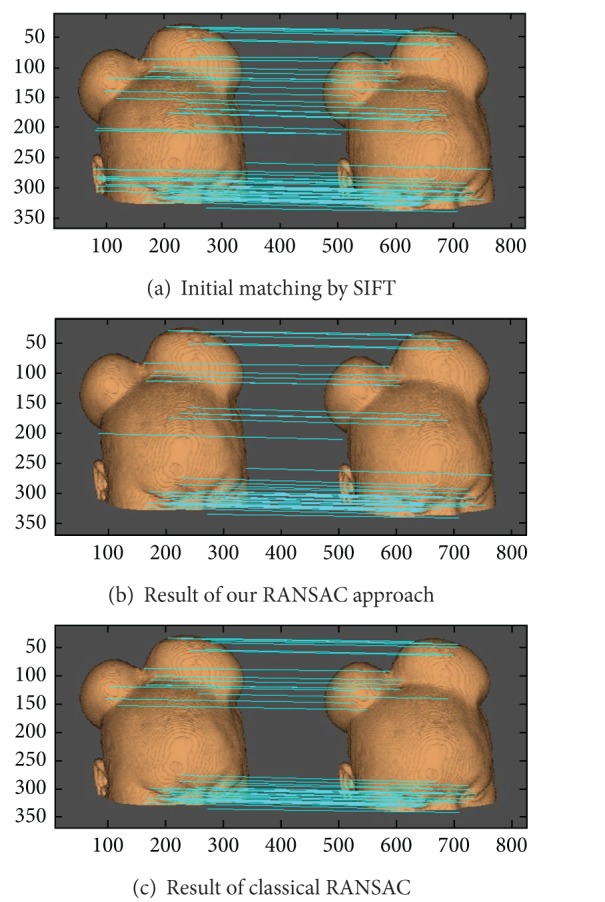
Results of the proposed method and classical RANSAC for correspondences based on SIFT.

Currently, SIFT is a popular and reliable algorithmto detect and describe local features in images. However, the initial matching by SIFT still exists in outliers. In this section, this paper uses the proposed approach to reject the outliers for the initial corresponding based on SIFT. The object is a model of scalp, which is usually used in biomedical modeling. The results are shown in Figure 4.  Figure 4(a) is the result of initial matching by SIFT, and the number of pairs is 68.  Figure 4(b) shows the result of our proposed RANSAC, the number of inliers is 50, and iteration times are 14.  Figure 4(c) illuminates the result of classical RANSAC in the same experimental condition, the number of inliers is 42, and iteration times are 31.

From the comparison results in Figure 4, it can be found that our method is more effective for outlier rejection. Moreover, the iteration times are reduced to almost 45%. It is the most important benefit of our approach. 

In conclusion, this paper argues that our method can be generally used in outlier rejection, no matter which kind of feature is used. Moreover, the preprocessing model is adaptive for the condition of low-matching rate. 

### 4.3. Analysis

**Figure 5:**
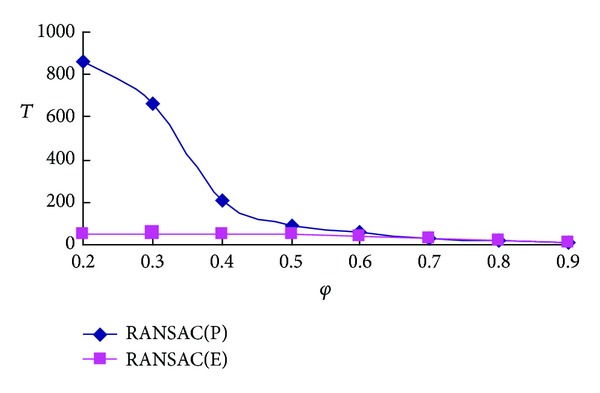
The number of iterations for RANSAC in set *E* and set *P* at the condition of different initial matching rates,  *T* represents the iteration time of RANSAC, and *φ* means the initial matching rate.

As is shown above, the proposed RANSAC succeeds in reducing the iteration times. Our framework's success owes to the preprocessing model, which is effective for selecting the reliable corresponding pairs.  Figure 5 illustrates the comparison of iteration times operating RANSAC in subset *E* and set *P*. It is obvious that there are huge differences especially when the initial matching rate is low. The main reason of the differences is that the elements of set *E* are much more reliable and with less scale. Through experimental statistics, it can be found that in the case of *φ* ≤ 0.6, the proposed RANSAC needs much less iterations than direct RANSAC processing does. While if the condition of *φ* is selected in 0.6 ≤ *φ* ≤ 0.9, the two methods usually have the same time complexity. Therefore, our model is beneficial to screen a reliable matching set *E* from the initial set *P* with lower matching rate *φ* and can reduce the followup of RANSAC iterations successfully.

## 5. Conclusion

In this paper, a novel framework was presented for improving RANSAC's efficiency in geometric matching applications. The improved RANSAC is based on Preprocessing Model that lets RANSAC operate on a reduced set of more confident correspondences with a higher inlier ratio. Compared with classic screening model, this model is simpler and efficient in implement, especially in the case of low-initial matching rate. The experimental results show that our approach can reduce much more iteration times especially when the initial matching rate is lower than 60%. In addition, the experiments were operated on two current features: Harris and SIFT. Therefore, it can be concluded that the proposed RANSAC framework is applicable.

In conclusion, this paper makes the following contributions: (1) this paper proposed a RANSAC framework which does not only rely on appearance but takes into account the quality of neighboring correspondences in the image space; (2) preprocessing model was introduced for selecting reduced set with higher inlier ratio, which improves runtime.

## Figures and Tables

**Algorithm 1:**
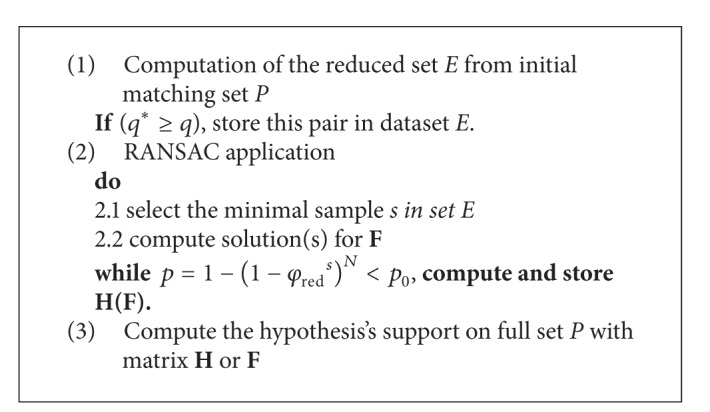
RANSAC with preprocessing model.
